# Preferential uptake of the non steroid anti-inflammatory drug diclofenac into inflamed tissues after a single oral dose in rats

**DOI:** 10.1186/1471-2210-9-5

**Published:** 2009-03-16

**Authors:** A Schweitzer, N Hasler-Nguyen, J Zijlstra

**Affiliations:** 1 Translational Sciences, Nonclinical PK/PD, Novartis Institutes for Biomedical Research, Novartis Pharma, Basel, Switzerland; 2 Department of Preclinical Development, Novartis Consumer Health, Rte Etraz, Nyon, Switzerland

## Abstract

**Background:**

Diclofenac is a nonsteroidal anti-inflammatory drug which is available as prescription (RX) and over-the-counter (OTC) medication for the systemic and topical treatment of painful and inflammatory conditions such as arthritis and back pain. This study was undertaken to investigate the distribution and retention of diclofenac and/or its metabolites in inflamed tissues, using the carrageenan-induced inflammation model and quantitative whole body autoradiography in rats.

**Methods:**

[^14^C]diclofenac sodium was administrated as a single 2 mg/kg oral dose 1 h after injection of carrageenan into one front and one hind footpads and subcutaneously into the dorsum of the neck of rats. A control animal received saline injections. Three carrageenan-treated rats and one control rat were sacrificed at 1, 4, 8, and 24 h after [^14^C]diclofenac sodium administration (total of 4 rats/time point). The carcasses were immediately snap-frozen and prepared for cryosectioning. Lengthwise whole-body sections (40 μm thick), including all major tissues, were obtained from different levels across the body. The tissue concentrations of total radiolabeled components were determined using quantitative autoradioluminography.

**Results:**

The radioactivity patterns demonstrated that diclofenac and/or its metabolites preferentially distributed into the inflamed tissues. In unharmed tissues the distribution was similar in control and treated animals. The exposure, based on the areas under the tissue concentration versus time (AUC_0-tlast_), was 26 and 53 fold higher in the inflamed neck and inflamed footpads of treated animals than in control rats; the exposures in unharmed tissues were similar in the treated and control rats, and the AUC_0-tlast _was 17 fold higher in the inflamed paws than in the non inflamed footpads of the carrageenan-treated rats. The higher exposure in the inflamed tissues may be explained partly to the fact that the elimination of total radiolabeled components from inflamed tissues (t_1/2 _= 6 h) appeared lower than from the corresponding unharmed tissues (t_1/2 _= 2 h).

**Conclusion:**

This animal study demonstrated that diclofenac and/or its metabolites were rapidly and preferentially taken up and retained in inflamed tissues. Although there were theoretical considerations that mildly acidic NSAID may show some preferential distribution in inflamed tissues there was no clear experimental proof for diclofenac until the present study.

## Background

Diclofenac belongs to the non steroidal anti-inflammatory class of drugs (NSAIDS) and is widely used in the treatment of moderate pain and inflammation, which are common symptoms of various diseases, including arthritis [1]. Diclofenac acts by inhibiting cyclooxygenases (COX), which is an enzyme that converts arachidonic acid into prostaglandins (PGs), thromboxanes and prostacyclins [2,3]. The COX-1 isoenzyme is constitutively expressed in all tissues [4] and its activation leads to the production of prostaglandins involved in maintenance of organs systems such as protection of the stomach wall or for the kidney function. Prostaglandin in the gastric and intestinal mucosa stimulates the secretion of mucus and bicarbonate which protect the gastrointestinal tract against acidity and digestive enzymes [5]. In contrast, COX-2 is almost undetectable in most tissues under normal physiological conditions [6-8], but is induced when there is a damage in the body, leading to the production of PGs. When proinflammatory cytokines are produced, COX-2 is expressed de novo in the inflamed tissue resulting in PGs formation, which mediates pain, fever and inflammation [9]. NSAID can inhibit both COX isoenzymes, but diclofenac demonstrates some selectivity towards COX-2 inhibition [10]. Efficacy of oral diclofenac intake for relieving pain and inflammation or fever has been demonstrated in several clinical studies with both low dose available over-the-counter or at higher dose available by prescription [11-15].

The present study aimed to investigate the relative distribution of diclofenac in inflamed and normal tissues in an animal model. It used radiolabeled drug that allowed the to visualize the temporal distribution of total components and to assess their concentrations in the various tissues by means of quantitative whole-body autoradiography (QWBA). Knowledge of the time course of the distribution of diclofenac in various organs is important in rationalizing the dosing schedule for various indications.

An inflammation model in rats was used in which rats were injected in the footpads with carrageenan, a mucopolysaccharide isolated from Irish sea moss. The model allows confirmation of the anti-inflammatory and analgesic effects of drugs such as acetylsalicylic acid, hydrocortisone or phenylbutazone at oral doses that are representative for their clinical use in rheumatic diseases [16]. Diclofenac, also, has been shown to be effective in this rat model after an oral single dose of 2 mg/kg, the dose that was selected for this pharmacokinetic study [17].

## Methods

### Chemicals

[^14^C]diclofenac sodium was provided by American Radiolabeled Compounds (101 Arc Dr, St Louis, MO 63146, USA) with a specific activity of 172 μCi/mg (50–60 mCi/mmole, 6.4 MBq/mg). Non-labelled diclofenac sodium supplied *in house *was used to dilute the radiolabeled material.

λ-carrageenan (article n° 22049-25G) was bought from Sigma-Aldrich (Buchs, Switzerland).

### Animals

Sixteen male pigmented Long Evans rats, 8 weeks old, weighing 230 ± 25 g, obtained from Charles River (Raleigh, NC, USA) were used. They had free access to rodent chow (Provimi Kliba SA, CH-4303 Kaiseraugst) and tap water during the entire study.

### Dose formulation

For dosing, 5.6 mg of cold diclofenac sodium and 4.4366 g of the radioactive solution above (corresponding to 2.5662 mg of [^14^C]diclofenac sodium) were dissolved in 40.038 g of 0.9% saline. The dose, 2 mg/kg b.w. of [^14^C]diclofenac sodium which has been reported to be the ED_40 _in this animal model [17], was given p.o. by gavage at 10 mL/kg in 0.9% saline. The radioactive dose was 4.11 MBq/kg b.w.

### Experimental design

Twelve rats were subjected to an acute local inflammatory reaction by subplantar injection with 0.1 mL of 1% w/v of carrageenan in 0.9% saline into the left front footpad pad and the left hind footpad and into the nape of the neck (0.4 ml of the solution and 0.4 ml air given subcutaneously). The other 4 rats (control group) received 0.9% saline into the same sites and at same volumes. The animals were slightly anesthetized with a 3% isoflurane/oxygen mixture before the injection.

One hour after carrageenan or saline injection, all animals received a single 2 mg/kg oral dose of [^14^C]diclofenac sodium. Blood (50 μL) was sampled from each animal shortly prior to sacrifice by sublingual puncture for QWBA validation purposes.

### Liquid scintillation counting (LSC)

Radioactivity in aliquots of the [^14^C]diclofenac sodium administration solution and of blood was determined by LSC in a Liquid Scintillation System 2500 TR (Packard Instr. Co., Meriden, CT, USA). For quench correction, the external standard ratio method was used. Quench correction curves were established by means of sealed standards (Packard Instr. Co.). Samples were prepared for analysis as described [18]. The limit of detection (LOD) for determination of radioactivity was defined as the 1.8-fold of the total background count.

### Quantitative whole-body autoradioluminography (QWBA)

Three treated rats and one control rat were used per time point. They were euthanized with an overdose of isoflurane at 1, 4, 8, and 24 h after dosing and thereafter immediately deep-frozen for approx. 30 min into an n-hexane/dry ice mixture kept at -70°C. The carcasses were rapidly shaven and the paws removed. The carcasses and the paws were then stored at a temperature below -20°C, and all subsequent procedures were performed at temperatures below -20°C to minimize diffusion of radiolabeled material in the tissues/matrices.

### Sectioning procedures

The frozen carcasses and paws were embedded in a mold on a microtome stage by adding an ice-cold aqueous solution of 2% sodium carboxymethylcellulose (CMC). The embedding block was frozen for approx. 30 min in an n-hexane/dry ice mixture at -70°C followed by temperature stabilization overnight in a -20°C freezer. The animals were sectioned using a CM 3600 PLC cryomicrotome (Leica Microsystems GmbH, D-Nussloch) according to the method of Ullberg [19]. Several sagittal 40 μm thick whole-body sections were taken at varying depths, based on sectioning of the requisite organs, tissues and body fluids. Similarly, several 40 μm thick sections were taken at varying depths from the paws. A block of ^14^C radiolabeled standards, prepared in blood and assayed by liquid scintillation counting, was sectioned in the same manner and on the same day as the rats were sectioned.

The sections were dehydrated in the cryomicrotome for 48 h at -30°C. Thereafter, they were labelled using radioactive ink specifying the test compound, radiolabel, protocol number, dose route, animal strain, time of sacrifice and number of section, resulting in a permanent label on each section and phosphor image autoradiogram.

### Imaging procedure

The whole-body autoradiograms were obtained by means of the autoradioluminography method [20]. Tissue/matrix and standard sections were placed in direct contact with Fuji BASIII Imaging plates (Fuji Photo Film Co. Ltd., J-Tokyo) for 3 days at room temperature in a lead shielded box in order to minimize the increase of the background signal. The duration of the exposure was chosen to allow detection of approx. 1 dpm/mg. At the end of the exposure, the sections were separated from the plates, and the plates were first kept for 3–5 min in the dark. They were then transferred into a Fuji BAS 2000 TR phosphor-imager (Fuji Photo Film Co. Ltd.) and scanned at a 100 μm scanning step with a 1024 gradation to produce an autoradiogram.

For reporting, selected sections were re-exposed onto SR screens (Perkin Elmer, formerly Packard Instr., Meriden, CN, USA) for 3 days at room temperature and scanned at a 42 μm scanning step in a ^®^Cyclone (Packard Instr.) phosphor-imager. The image files were processed using the Adobe Photoshop Elements 2.0 software (Adobe Systems Incorporated, San Jose, California, USA).

### Determination of the tissue/matrix concentrations of total radiolabeled components

The concentrations of total radiolabeled components in the tissues/matrices were determined by comparative densitometry and digital analysis of the autoradiogram. The radioactivity concentrations were calculated from the curve generated from the calibration samples present on the image plate and were expressed as nanomoles/gram of tissue/matrix [21]. The resulting photo-stimulated light data files were corrected by subtracting the background and processed electronically with the help of a MCID/Elite (Version 6.0) image analyzer (Imaging Research, St. Catherines, Ontario, Canada). The calibration standards were prepared from fresh whole rat blood and a stock solution of a [^14^C]-labelled compound. Up to seven calibration concentrations were prepared at concentrations of 2–4000 dpm/mg for ^14^C which encompass the range of concentrations typically observed in a standard whole-body autoradiography study. To assess the actual concentrations in the calibration standard samples, duplicate 100 μL samples of spiked blood were counted for total radioactivity in a liquid scintillation counter. These values were defined as the actual concentrations and used to generate a calibration curve during digital analysis.

Quality control (section thickness homogeneity and reproducibility checks) was performed by analyzing the radioactivity concentration in the liver on each section. In addition, the concentrations of radioactivity as determined by QWBA in blood were compared to the corresponding ones determined by LSC (blood samples collected from each animal shortly prior to sacrifice).

The limit of detection (LOD) was set equal to the mean of background (n = 10) + 3 SD (standard deviations of the individual values). The quantification limit (LOQ) was empirically taken as 3•LOD. The size of the areas used to determine the background value was normalized to that of the blood standards used to establish the calibration curve. Under the conditions of this study, the LOD and LOQ amounted to 0.034 and 0.10 nmol/g, respectively.

### Tissue/matrix distribution of radioactivity

The peak concentrations of radioactivity C_max _and time of peak concentration t_max _were recorded as observed. The AUC(0-t_last_) and t_1/2 _were calculated for those tissues/matrices where sufficient quantifiable data points were available. The AUC(0-t_last_) were calculated using the linear trapezoidal rule. The t_1/2 _was taken as ln(2)/λ_z _where λ_z _(terminal elimination rate constant) was the slope of the log linear line from the at least 3 last measurable data points. All calculations were performed using the computer program WinNonlin^® ^(Professional 5.0) (Pharsight Corp., Mountain View, CA, USA). The ratios of tissue/matrix to blood C_max _and AUC(0-t_last_) values were also reported where possible.

### Residual radioactivity in the body at 24 h after dosing

In order to estimate the overall residual radioactivity in the body at 24 h after dosing, the mean concentrations of total radiolabeled components over the entire section of at least 6 sections per rat (one per sectioning level) were determined as described above and converted into percent of the administered dose by taking into account the weight of the animals, the specific activity of the administration formulations, the volume administered and the actual doses.

## Results

### Distribution in tissues

**Figure 1:**
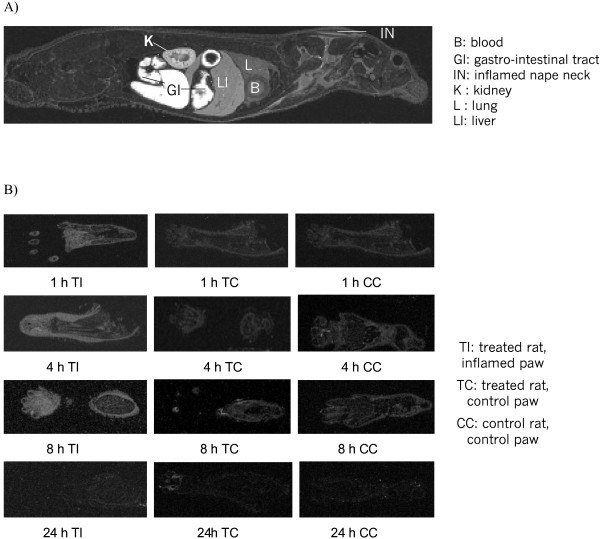
**Selected whole-body autoradiograms of treated rats at 4 h in the whole animal (A), and in the injected footpads of the treated and the control animal (B) after a 2 mg/kg oral dose of [^14^C]diclofenac sodium**. The white areas correspond to the high radioactivity concentrations.

Analysis of the whole body autoradiograms as shown in Figure 1 allowed to determine concentrations of diclofenac related material in the various tissues. Table 1 summarizes the values obtained at 1, 4, 8, and 24 h after dosing of [^14^C]diclofenac sodium.

**Table 1:** Tissue concentrations of total radiolabeled components at 1, 4, 8 and 24 h after oral administration of [^14^C]diclofenac sodium at a dose of 2 mg/kg to male rats injected with carrageenan (T) or saline (C) into the left front footpad and the left hind paw.

	Concentration of total radiolabeled components (nmol/g)							
	1 hour		4 hours		8 hours		24 hours	

Tissue	T	C	T	C	T	C	T	C

Injection site nape neck	0.79 ± 0.12	0.18	1.20 ± 0.30	tc	1.30 ± 0.10	tc	0.20 ± 0.04	nd

Untreated footpads	0.16 ± 0.04	0.20	0.15 ± 0.10	0.20	tc	nd	nd	nd

Injection site footpads	1.00 ± 0.23	0.12	1.30 ± 0.5	nd	0.84 ± 0.10	nd	tc	nd

Adrenal (cortex)	1.00 ± 0.32	0.29	0.94 ± 0.40	0.87	0.61 ± 0.10	0.59	tc	nd

Bile	34.00 ± 6.25	20.00	34.00 ± 16.40	12.00	15.00 ± 3.40	7.50	1.60 ± 0.22	1.40

Blood	1.60 ± 0.27	1.00	2.20 ± 1.10	1.90	1.50 ± 0.20	1.20	0.15 ± 0.02	0.11

Bone mineral	tc	tc	tc	tc	tc	tc	na	na

Brain	tc	tc	tc	tc	tc	na	tc	na

Cartilage	tc	tc	tc	tc	tc	na	na	na

Eye (lens)	tc	tc	tc	tc	tc	tc	tc	na

Eye (vitreous body)	tc	tc	tc	tc	tc	tc	tc	na

Fat (brown)	0.69 ± 0.13	0.45	0.73 ± 0.30	0.81	0.46 ± 0.10	0.32	tc	na

Fat (white)	tc	tc	0.12	0.15	tc	tc	tc	na

Heart	0.86 ± 0.13	0.52	1.00 ± 0.50	1.00	0.68 ± 0.10	0.56	tc	na

Kidney	11.00 ± 1.81	5.20	17.0 ± 5.50	11.00	9.10 ± 1.60	7.9	1.70 ± 0.09	1.20

Liver	4.50 ± 0.85	3.50	5.40 ± 1.10	3.90	3.0 ± 0.60	2.1	0.46 ± 0.05	0.31

Lung	1.20 ± 0.14	0.69	1.50 ± 0.70	1.30	1.00 ± 0.10	1.0	0.12 ± 0.01	0.10

Muscle	0.17 ± 0.04	0.11	0.22 ± 0.10	0.16	0.13 ± 0.01	0.12	tc	T

Oesophagus	5.00 ± 1.03	9.20	3.50 ± 1.43	1.50	0.33 ± 0.15	0.39	na	na

Penis	0.95 ± 0.15	0.42	7.00 ± 5.27	1.10	1.20 ± 0.11	0.65	0.27 ± 0.08	0.17

Seminal vesicle	tc	tc	tc	tc	tc	tc	na	nd

Spinal Cord	tc	tc	tc	tc	tc	tc	na	nd

Thyroid glands	0.86 ± 0.21	0.59	0.58 ± 0.11	0.60	0.46 ± 0.03	0.44	tc	tc

Tooth pulp	1.20 ± 0.03	0.76	1.20 ± 0.23	1.00	1.00 ± 0.12	0.98	0.13 ± 0.04	0.10

na: not applicable, nd: not detected (< limit of detection 0.034 nmol/g), tc: Traces (between limit of detection and limit of quantification (0.10 nmol/g))

Values are mean ± standard deviation (SD)for the T (n = 3) and mean value in C (n = 1).

At 1 h after dosing, the distribution of total radiolabeled components in the treated rats was mainly vascular, with concentrations higher than in blood (mean of 1.6 nmol/g) being found only in the bile, kidney, oesophagus and liver (means of 34.0 to 4.5 nmol/g). Concentrations around 1 nmol/g were found in the lung, tooth pulp, adrenal cortex, penis, heart, and thyroid gland (means of 1.2 to 0.86 nmol/g), as well as in the inflamed footpads (1.0 nmol/g). The mean concentrations of total radiolabeled components in the neck and non-inflamed footpads amounted to 0.79 nmol/g and 0.16 nmol/g, respectively. The lowest concentrations of total radiolabeled components were detected in the bone mineral, brain, cartilage, lens, vitreous body, white fat, seminal vesicle, and spinal chord (traces). No radioactivity was detected in the blood vessel wall and hair (LOD: 0.034 nmol/g).

The distribution pattern and overall concentrations in the treated rats at 4 h and 8 h after dosing were quite similar to those at 1 h after dosing, with the exception of the high concentration found at 4 h after dosing in the penis. At 4 h after dosing, the bile, kidney, liver, penis, and oesophagus (means of 34 to 3.5 nmol/g) showed higher concentrations than blood. The mean concentrations of total radiolabeled components in the inflamed nape neck inflamed footpads and non-inflamed footpads amounted to 1.2, 1.3 and 0.15 nmol/g, respectively at 4 h after dosing, and to 1.3 and 0.84 nmol/g and to traces, respectively, at 8 h after dosing.

At 24 h after dosing, concentrations of total radiolabeled components above the quantification limit (0.10 nmol/g) were found in the treated rats only in the neck (application site), bile, blood, kidney, liver, lung, penis, preputial gland, and tooth pulp (means of 1.7 to 0.12 nmol/g). The mean levels in the neck (application site) and inflamed footpads amounted to 0.20 nmol/g and to traces, respectively. No radioactivity was detected in the control footpads.

The distribution patterns of radioactivity in the control rats were similar to those in the treated animals, and are therefore not described in detail. However the overall tissue concentrations in the control rats appeared to be slightly lower than in the treated animals.

### Pharmacokinetics parameters

As shown in Table 2, the maximal concentration (C_max_) was reached within 4 to 8 h (t_max_) after dosing in the inflamed footpads and the nape of the neck while in the corresponding unharmed tissues t_max _was at 1 h. Compound related material was eliminated from most tissues with a half-life (t_1/2_) of about 6 h. An even more rapid elimination, with t_1/2 _of about 2 h, was observed from control footpads of treated rats and from corresponding unharmed tissues.

**Table 2:** Pharmacokinetic parameters of total radiolabeled components in selected tissues/matrices, calculated from the radioactivity concentrations determined by QWBA after a 2 mg/kg oral dose of [^14^C]diclofenac sodium in treated rats (n = 3 animals/time point) and in control rats (n = 1 animal/time point).

Treated rats (Carrageenan injected)	t_max _(h)	C_max _(nmol/g)	AUC(0-t_last_) (h*(nmol/g))	t_last _(h)	t_1/2 _(h)
Control footpads	1	0.16	0.92	8	1.80

Inflamed footpads	4	1.30	16.00	24	5.30

Inflamed nape neck	8	1.30	20.00	24	7.10

Blood	4	2.20	27.00	24	5.10

Kidney (cortex)	4	9.80	105.00	24	5.20

Liver	4	5.40	62.00	24	5.70

Control rats (Saline injected)	t_max _(h)	C_max _(nmol/g)	AUC(0-t_last_) (h*(nmol/g))	t_last _(h)	t_1/2 _(h)

Control footpads	1	0.20	1.1	8	2.60

Injected footpads	1	0.12	0.30	4	1.60

Injected nape neck	1	0.18	0.77	8	2.90

Blood	4	1.90	22.00	24	4.90

Kidney (cortex)	4	8.20	88.00	24	5.30

Liver	4	4.00	45.00	24	5.50

### Uptake of total radiolabeled components into the inflamed tissues

Figure 1B and data from Table 3 clearly show that the concentrations of total radiolabeled components were higher in the inflamed footpads and inflamed nape neck of the treated animals than in the control animals. The ratio of the AUC_0-tlast _between the treated and control rats amounted to 26 and 53 for the inflamed neck and footpads, respectively, whereas in others tissues this ratio ranged between 0.8 and 1.4. When compared in the same animal the AUC in the inflamed footpads was 17 fold higher than in the non inflamed footpads (Figure 1B; Table 3).

**Table 3:** AUC ratios in selected tissues of treated (T; carrageenan injected) and control (C; saline injected) rats after a 2 mg/kg p.o. dose of [^14^C]diclofenac sodium.

AUC(0-t_last_) h*(nmol/g)	Treated group T	Control group C	Ratio T/C
Control footpads	0.92*	1.10	0.82

Injected footpads	16.00*	0.30	53.00

Injected nape neck	20.00	0.77	26.00

Blood	27.00	22.00	1.20

Kidney (cortex)	105.00	88.00	1.20

Liver	62.00	45.00	1.40

The ratio in the treated group between the injected footpads and the control footpads (*) amounts to 17.

### Residual radioactivity in the body

Residual radioactivity in the body at 24 h after dosing amounted to a mean of 1.4 nmol/g in the treated rats and to 1.0 nmol/g in the control rat, corresponding to approx. 22% and 19% of the dose, respectively.

## Discussion

The overall distribution patterns of total radiolabeled components in the unharmed tissues were comparable in animals injected with saline or with carrageenan, at all time points investigated. The highest concentrations throughout the study were found at 1 and 4 h after dosing in the bile, kidney and liver, showing the biliary and renal excretion of this compound and its metabolites, in agreement with previous findings [22]. The concentration of total radiolabeled components in the oesophagus in animals sacrificed at 1 hour after dosing was quite high, which may be related to the oral dosing procedure.

[^14^C]diclofenac and/or its metabolites had the tendency to reach higher concentrations in the inflamed footpads and inflamed nape neck in the carrageenan injected animals when compared with the non-inflamed footpads from either the same animal or the control animal. This latter was mainly used to evaluate if the saline injection, alone, might induce inflammation. However the results of Table 1 exclude this assumption as accumulation of radiolabeled components was similar between saline injected and non injected footpads from either the same control animal (C) or from the treated animal (T).

The highest concentration of total radiolabeled components in the inflamed footpads was observed 4 h after injection of carrageenan. This is in accordance with a previous publication reporting that in this acute inflammation model COX-2 expression peaked at 1 h with maximal PGs production after 2 to 4 h as a result of the increased cyclooxygenases activity [23]. Depending of the site of local carrageenan injection, the kinetic profile of the radioactivity concentration was slightly different, which correlates with a previous study showing that carrageenan elicits response which differs over time when injected at different sites in the same rat [24].

**Figure 2:**
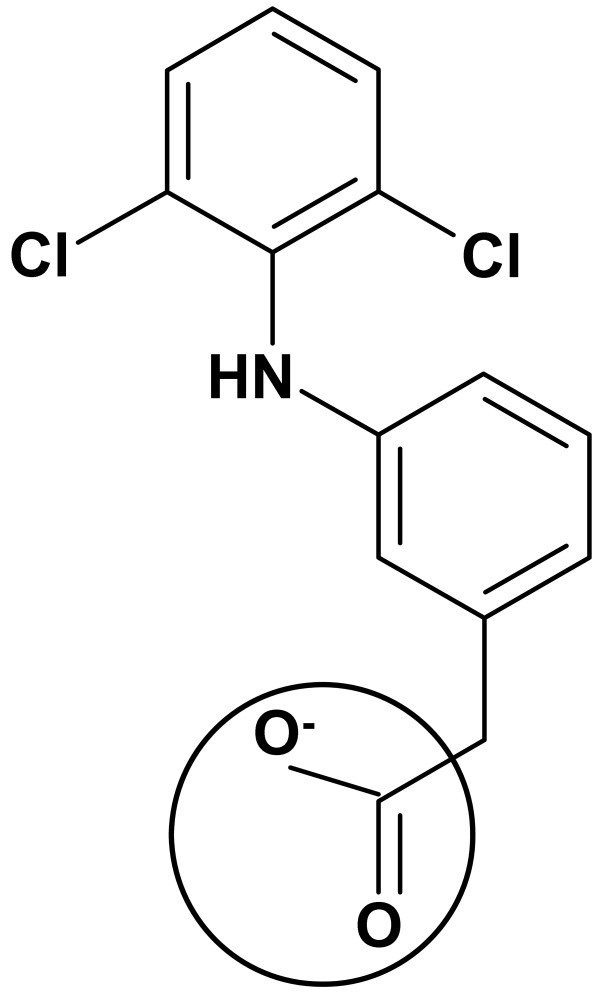
**Chemical structure of diclofenac sodium which acidic group is circled**.

Using autoradiography techniques, several studies have shown that acidic NSAIDs preferentially accumulate in acidic compartment, such as the stomach [25], kidney [25] and inflamed tissues [26,27]. As shown in Figure 2 diclofenac sodium with its carboxylic acid group has a pKa of 4.6 and belongs to the class of weak acidic drugs, which may show increased lipophilicity in acidic compartment, leading to higher concentrations in cell membranes [27,28]. In addition, the weak acidic properties of NSAID as well as the bipolar lipophilic and hydrophilic physico-chemistry of these drugs enable them to bind to a high degree to plasma proteins *in vivo *[29]. This is due to the high concentration of the fatty acid transporting protein albumin [30]. Subsequently these drugs are distributed mainly in the blood compartment. Therefore it is suggested that the drug would be found in highly vascularised tissues such as liver, lung, penis, preputial gland, tooth pulp, which is supported by the current data (Table 1). In contrast, only traces of radioactivity were detected in eyes, brain, seminal vesicle, and the spinal cord, as well as the bone mineral and the cartilage which lack vascularisation. Higher accumulation of total radiolabeled components was detected in the kidney, which might not be dependent on the blood flow because plasma proteins are not filtrated through the glomerulus. It would be assumed that free diclofenac and its metabolites might be taken up by the kidney through organic anion transporter (OAT) on membrane of the renal proximal tubular cells [31]. Excretion of drugs is mainly through the hepatobiliary or the renal routes but both pathways are complementary each other. Drugs bound to plasma proteins are eliminated through the bile while the kidney eliminates more hydrophilic compounds and generally less bound to proteins. Therefore the difference in physicochemical properties of diclofenac and its metabolites might result in difference in affinity for membrane transporters or for plasma proteins. This is translated in the present study by the observation of an increased concentration of radiolabeled components in the kidney and the bile (Table 1).

In the non inflamed part of the neck and the control footpads, blood vessels density is low and no inflammation was induced by the remote injection of carrageenan. Subsequently the clearance of the drug or its related compound from these tissues was rapid as shown in Table 2. The present data consolidate the observation of Brune [27], reporting that the capillary damage due to inflammation results in plasma protein accumulation and concomitantly accumulation of the drug bound to these proteins, causing an elevated drug concentration around the leaky capillary. This raises the question whether this protein/drug complex might be efficient in inhibiting the COX-2 enzyme. Recent results from an *in vitro *study demonstrated that increasing plasma protein did not affect the potency of diclofenac to inhibit the COX-2 activity [32] which might support the view that the anti-inflammatory effect of protein-bound diclofenac is comparable to the free drug. Brune has proposed that the local acid microenvironment caused by inflammation might favour uptake of the neutral chemical form of diclofenac into the cell membrane and into the cytoplasm of cell, where binding to COX-2 enzymes may occur. [33].

## Conclusion

This study allowed to assess the concentrations of diclofenac and/or its metabolites in the tissues up to 24 hour after oral administration, emphasizing that binding of the drug to plasma proteins, hence tissue perfusion, is a key factor in distribution of diclofenac to its site of action or to the hepato-biliary system. By contrast, metabolism of diclofenac, resulting in more hydrophilic compounds that are less bound to plasma proteins, might lead in higher uptake in the kidney by membrane transporter proteins.

Though there were theoretical considerations that mildly acidic NSAID may show some preferential distribution in inflamed tissues, there was no clear experimental proof for diclofenac until the present study.

## Abbreviations

COX: cyclooxygenases; NSAID: non steroid anti inflammatory drug; PGs: prostaglandins E2; QWBA: quantitative whole-body autoradiography.

## Competing interests

AS, NHN and JZ are employees of Novartis, which produces and distributes the diclofenac therapy products.

## Authors' contributions

AS conducted and managed the study and analyzed the imaging data, NHN drafted the manuscript. JZ participated in the design and coordination of the project. All authors cooperatively designed the project and discussed data interpretation. All authors participated in critical editing of the manuscript.
